# Investigating Environmental Features and Network Interdependencies in Network Formation: A Study of Collaborative, Community-Based Watershed Restoration

**DOI:** 10.1007/s00267-026-02433-0

**Published:** 2026-04-02

**Authors:** Nicholas Oesterling

**Affiliations:** Syracuse University – Maxwell School of Citizenship and Public Affairs 400 Eggers Hall, Syracuse, NY USA

## Abstract

Public policies, particularly those addressing environmental problems, increasingly depend on the work of networks of governmental and non-governmental actors to achieve policy goals, making an understanding of the formation of these networks imperative for the study and practice of environmental management. Past scholarship often examines networks for collaborative policymaking or interorganizational planning, with less attention being given to networks in which participants take hands-on action together to tackle public problems “on the ground”. Yet, these action-oriented networks can be crucial for policy implementation. This paper responds to this knowledge gap by analyzing the formation of purpose-oriented networks (PONs) by organizational actors for collaborative restoration projects in Oregon watersheds. PONs form when actors consciously convene under some shared affiliation to work together, potentially through cross-sector collaboration, to accomplish some shared goal(s). Through regression analyses using over two decades of data on thousands of PONs and their participants, I investigate factors that may influence when and where these networks form. I find PON formation may be catalyzed by increased resource contributions to previously-formed networks, the presence of more saturated network environments (i.e., environments where more networks developed in the prior year), and stakeholders’ prior participation in multiple networks simultaneously. Moreover, network formation could be diminished when PONs formed in the previous year take longer to complete their work. My findings offer researchers and practitioners novel insights into PON creation and draw attention to how the constitutive dimensions of these networks may have implications for their developmental dynamics.

## Introduction

In recent years, policymakers have begun enacting policies that rely on governmental and non-governmental actors working together across jurisdictional and sectoral boundaries to address complex public problems (Emerson and Nabatchi 2015; Siddiki et al. 2022; Ulibarri et al. 2023). One form of these cross-boundary arrangements that has increasingly gained the attention of policymakers and researchers is purpose-oriented networks (PONS) – bounded groups of three or more actors (e.g., individuals, organizations) working together under some self-referencing affiliation (e.g., a group name) to achieve some shared purpose(s) (Nowell and Milward 2022). In developing a taxonomy of networks in public management and policy, Nowell and Milward (2022) note that PONs are a class of networks that occur in a variety of forms, including cross-sector collaborations or partnerships (Bryson et al. 2006; 2015) and collaborative governance regimes (Emerson and Nabatchi 2015; Emerson et al. 2012).

In this study, I focus on PONs that convene organizational actors from different sectors or jurisdictions to solve some complex problem(s) through collaboration in which network members determine an agreed-upon course of action, pool resources, and jointly implement some solution(s) (Bryson et al. 2006; 2015; Gray 1985; Margerum 2011). PONs for such collaboration have steadily emerged as vehicles for policymaking, planning for policy goal achievement, and on-the-ground policy implementation (Margerum 2011; Nowell and Milward 2022; Provan and Kenis 2008). Furthermore, these PONs can facilitate broader collaborative governance processes and structures in which actors engage in an ongoing and evolving interactive system of collaboration for public decision-making around achieving the goals of public policies (Ansell and Gash 2008; Emerson and Nabatchi 2015; Emerson et al. 2012; Nowell and Milward 2022).

Although a substantive body of work examines how PONs for cross-boundary collaboration operate (e.g., how members work together), it is imperative that we also understand how these networks form, given the increasing reliance of public officials on these arrangements. Moreover, prior research, particularly in environmental management, predominantly focuses on networks at what Margerum (2011) identifies as the policy level (i.e., deliberating for the design, passage, and enactment of policies) or the organizational level (i.e., integrating organizations’ strategies or plans). Less attention has been given to networks at the action level (Margerum 2011), where actors work together for the completion of hands-on, on-the-ground activities (e.g., tree planting, river clean-up) to implement public policies. Yet, action-level efforts can serve as meaningful pathways for non-governmental stakeholders to directly contribute to addressing public problems (Rickenbach and Reed 2002), especially those that governments have not, or cannot, tackle effectively on their own (e.g., nonpoint source pollution) (Margerum 2008).

I respond to these research gaps through an investigation of influences on the formation of PONs for cross-sector collaborations desired by public policies. More specifically, I focus on the formation of action-level PONs for the completion of collaborative restoration projects by stakeholder organizations in Oregon watersheds. These project-based PONs form under the guidance, incentives, and other tools of state policies, predominantly the Oregon Plan for Salmon and Watersheds (OPSW), designed to foster cross-sector and interjurisdictional collaboration among watershed stakeholders. Oregon policymakers rolled out the OPSW in response to the consequences of decades of detrimental actions (e.g., agricultural runoff, industrial pollution) in the U.S. Pacific Northwest. Using over two decades of data, I evaluate potential influences on the formation of over 10,000 PONs created under the auspices of the OPSW.

To guide this inquiry, I advance two research questions. First, are influential factors in the creation of policy- or organizational-level PONs meaningful for action-level PON formation? Second, do inter-PON dynamics, such as prior network creation or overlapping membership between networks, shape new PON creation? To address these questions, I cross-pollinate theoretical perspectives in policy process and public management scholarship along with research on networks and cross-sector collaboration to contribute to our knowledge of PON formation for policy implementation. Moving forward in this article, I review fundamental scholarship on PONs and use earlier research on watershed collaborations and network interdependencies to develop hypotheses around the formation of project-based PONs for watershed restoration in Oregon. I then describe my research case, data, and analytical approach in more detail before reporting my findings, identifying avenues for future research, and discussing the study’s relevance for scholars and practitioners.

## Literature Review

### Purpose-Oriented Networks and Their Network Domains

A purpose-oriented network (PON) is a group of three or more actors, often organizations, that exhibits three fundamental conditions: (1) the network refers to itself as a collective entity (e.g., through a group name); (2) the network has bounded membership; and (3) network members collectively recognize and consciously engage with some common purpose(s) for which the network exists (Nowell and Milward 2022). Although the PON classification is relatively new in network research (Carboni et al. 2019; Nowell and Kenis 2019; Nowell and Milward 2022), these networks have been studied for quite some time (Carboni et al. 2019; Nowell and Milward 2022). Nowell and Milward (2022) identify PONs as a class of networks encompassing network labels used in earlier studies, including “goal-directed networks” containing autonomous organizations working to achieve individual goals and some collective goal(s) (Saz-Carranza and Ospina 2011), “whole networks” comprised of organizations connected through ties enabling the realization of a common goal (Provan et al. 2007), and “public management networks” operating as collaborative structures bringing together governmental and nongovernmental actors to address shared problems (Agranoff 2007).

Furthermore, Nowell and Milward (2022) posit that PONs can operate as cross-sector collaborations (Bryson et al. 2006; 2015) through “the linking or sharing of information, resources, activities, and capabilities by organizations from two or more sectors that could not be achieved by organizations in one sector separately” (Bryson et al. 2006, p. 44). The “recognized interdependence” (Bryson et al. 2015, p. 651) of participants is a critical catalyst of cross-sector collaboration (Bryson et al. 2015; Gray 1985). PON formation is also driven by some shared purpose(s) and recognized need for joint effort that network members acknowledge and accept as the reason(s) that the network forms (Carboni et al. 2019; Isett et al. 2011; Nowell and Milward 2022; Provan and Milward 2008). Thus, although network members remain autonomous, they contribute to some “joint production function” (Carboni et al. 2019, p. 214) that facilitates the achievement of the shared purpose around which a PON’s members consciously convene and collaborate (Carboni et al. 2019; Isett and Provan 2005; Nowell and Milward 2022).

While actors’ recognition of their interdependence is a driver of network development, other factors may also shape actors’ decision-making around network formation, such as trust, partner similarity, and expected gains (Siciliano et al. 2021). Much of this extant scholarship often examines network development from an internally-focused point of view (Nowell and Kenis 2019; Nowell et al. 2019; Rethemeyer and Hatmaker 2008). Owing to this internal focus, we know quite a bit about how compositional or functional mechanisms may affect network creation, but more research is needed to identify and unpack the ramifications of external factors (e.g., resource interdependencies) (Nowell and Albrecht 2024; Nowell and Kenis 2019; Rethemeyer and Hatmaker 2008).

To more tractably study PONs from such an externally-oriented perspective, Nowell, Hano, and Yang (2019) draw on open system organizational theories (Katz and Kahn 1978) and organizational ecology (Hannan and Freeman 1977) to develop the concept of a network domain. They define a network domain as a population of PONs sharing a common mission focus and operating within the same geographic area (Nowell et al. 2019). Prior studies of network domains use county jurisdictions as boundaries for examining PONs’ environments (e.g., Nowell and Albrecht 2024; Nowell et al. 2019), but this boundary decision ultimately stems from a study’s context and a researcher’s aims for their work (Nowell and Milward 2022; Taylor and Nowell 2024). In this study, I use watershed subbasins to delineate network domain boundaries.

Through its identification of a specific set of PONs with some combination of shared purpose and proximity, the network domain serves as a useful analytical boundary for investigating PON formation in the context of networks’ environments (Nowell and Albrecht 2024; Nowell et al. 2019). By operationalizing network domains as watershed subbasins, I am able to analyze what aspects of these domains are tied to PON formation within them. In this analysis, I specifically focus on environmental features of network domains and on interdependencies between PONs within the same domains. In the following sub-sections, I advance hypotheses about potential relationships between network domain characteristics and PON formation.

### Environmental Features and PON Formation

Prior work on the formation of groups for collaborative efforts in watersheds, including networks for policymaking and planning, broadly identifies influences under three themes: (1) resource quality and problem severity, (2) institutions and policy actors, and (3) community and/or stakeholder capacities (Margerum 2011; Sabatier et al. 2005). Each of these influences can potentially affect PON formation. Beginning with resource quality and problem severity, poor water resource conditions are highlighted as potential catalysts for collaborations that address these issues (Diaz-Kope and Miller-Stevens 2015; Diaz-Kope and Morris 2022; Kharel et al. 2018). Relatedly, as the ramifications of declining resource conditions become more apparent or tangible, watershed stakeholders tend to be more strongly motivated to participate in collaborative efforts (Church and Prokopy 2017; Diaz-Kope and Miller-Stevens 2015; Margerum 2011). In accordance with these findings, I advance the following hypothesis:

**Hypothesis #1a:** Greater PON formation will occur in network domains where water resource conditions were previously worse.

In addition to resource conditions, scholars note the influence of institutions (e.g., laws, rules, interlocal agreements) and the activities of actors with key roles in policy implementation on the formation of watershed collaborations for policymaking and interorganizational planning. The institutions comprising policy tools like incentives and rules can shape groups’ membership, internal decision-making, and day-to-day operations (Diaz-Kope and Morris 2022; Emerson and Nabatchi 2015; Olivier and Schlager 2022). Furthermore, public policies are increasingly prescribing certain actors, often government agencies, with crucial roles in carrying out or supporting policy implementation (Ingold et al. 2021; Margerum 2011). The participation of these “policy-prescribed” actors can serve as drivers for the development and operation of watershed collaborations (Chaffin et al. 2015; Genskow 2009; Ulibarri et al. 2023). As such, I make the following hypothesis:

**Hypothesis #1b:** Greater PON formation will occur in network domains where key policy-prescribed actors participated more extensively in active (i.e., newly formed or ongoing) PONs in the prior year.

Along with resource conditions and the activity of policy-prescribed actors, various capacities (e.g., ecological systems, social ties, economic interlinkages) of the communities within watersheds may affect the formation of policy- or organizational-level collaborations (Johnson et al. 2017; Lubell 2004; Sabatier et al. 2005). Attributes like stakeholders’ financial and technical capital are thought to affect formation of, and participation in, watershed collaborations (Margerum 2011; Sabatier et al. 2005) as these groups often rely on a variety of participant-provided resources, including monetary offerings (e.g., grants, donations) and in-kind contributions (e.g., equipment, physical labor, topical expertise or consulting) (Chaffin et al. 2015; Koehler and Koontz 2008; Mehdi and Nabatchi 2023). Prior research highlights the potential ramifications of stakeholders’ resource capacities on their participation in environmental policymaking and planning networks (Angst et al. 2022; McLaughlin et al. 2022). Consequently, I advance the following hypothesis:

**Hypothesis #1c:** Greater PON formation will occur in network domains where, in the previous year, new PONs acquired more resources.

### Network Interdependencies and PON Formation

**Fig. 1:**
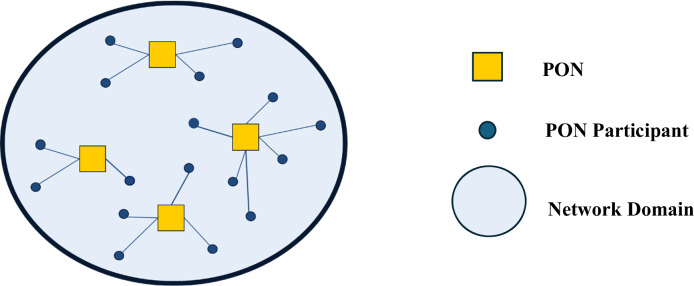
A Stylized Example of a Differentiated Network Domain

**Fig. 2:**
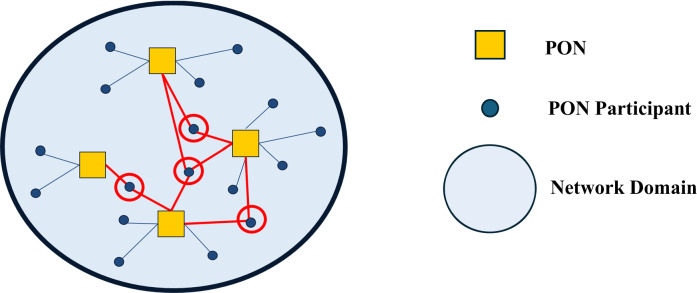
A stylized example of an undifferentiated network domain

As discussed earlier, Nowell, Hano, and Yang (2019) note that network analysts often tacitly assume that networks operate relatively independent of each other within a network domain whereby a network’s membership is effectively exclusive. Nowell, Hano, and Yang (2019) refer to the theoretical case in which no membership overlap exists between a domain’s PONs as a differentiated network domain (shown in Fig. 1). Alternatively, an undifferentiated network domain (shown in Fig. 2) contains PONs with some degree of shared membership (Nowell et al. 2019). Nowell, Hano, and Yang (2019) demonstrate the existence of undifferentiated domains and past research emphasizes the potential of an externally-focused perspective of networks that considers how the existence of undifferentiated network domains may enable more comprehensive understanding of network formation and performance (e.g., Nowell and Albrecht 2024; Nowell et al. 2019; Rethemeyer and Hatmaker 2008). Notably, Nowell and Kenis (2019) posit, and Nowell and Albrecht (2024) identify, that resource interdependencies can occur among PONs in an undifferentiated domain. The possibility of such interdependencies between a domain’s PONs is nontrivial. Resources are essential for network performance (Agranoff and McGuire 2001; Auschra and Sydow 2023; Nowell and Albrecht 2024; Provan and Milward 2001). Network members are, themselves, fundamental resources for a network, along with being the primary channel for other critical network resources including ideas, information, legitimacy, financial backing, or other capital (Nowell and Albrecht 2024; Provan and Milward 2001; Shropshire 2010).

In considering the potential ramifications of undifferentiated network domains, the saturation of a domain (i.e., the number of PONs operating within it) may affect the formation of new networks. Within undifferentiated domains, population dynamics can exist as new PONs may draw on the same pool of stakeholders and, by extension, other resources as existing PONs (Nowell and Albrecht 2024). These interdependencies could shape the extent to which new networks develop in a domain (Nowell and Albrecht 2024; Nowell et al. 2019; Rethemeyer and Hatmaker 2008). For instance, as more PONs form and saturate a domain, resources like network members can become scarcer, and acquiring these resources becomes more competitive (Baum and Oliver 1996; Baum and Singh 1994). Increases in a domain’s saturation and resource scarcity may affect the extent of network formation that it experiences in the future (Amburgey and Rao 1996). Nowell and Albrecht (2024) find support for this scenario in their study of network domains of healthcare PONs at two points in time, five years apart, with less saturated domains being more likely to experience increases in the total number of networks active within them. Considering these past insights, I advance the following hypothesis:

**Hypothesis #2a:** Less PON formation will occur in more saturated network domains, within which more PONs formed in the prior year.

Relatedly, as part of their examination of undifferentiated network domains, Nowell, Hano, and Yang (2019) leverage literature on interlocking corporate directorates to develop the concept of membership interlock for describing instances when an actor is simultaneously a member of two or more PONs in the same domain. Interlocks formed by PONs’ members could pose benefits or constraints for these actors and the networks in which they participate. On one hand, interlocks may serve as valuable assets to PONs and their members, connecting them to other networks and enabling new opportunities for learning and/or further collaboration (Angst et al. 2022; Fried et al. 2023; Mizruchi 1996; Shropshire 2010). On the other hand, as PONs’ members are frequently organizational actors with finite attention and resources, interlocks may disperse and effectively limit the amount of resources that a member can contribute to a given PON (Nowell and Albrecht 2024; Nowell et al. 2019; Provan and Milward 2001). Thus, a PON’s members, along with being vital resources for the network (Agranoff and McGuire 2001; Auschra and Sydow 2023), may also tie the network to other PONs in its domain. These inter-PON connections may shape network activity (e.g., dissolution, creation) within a domain (Nowell and Albrecht 2024; Nowell et al. 2019; Nowell and Kenis 2019). Nowell and Albrecht (2024) find a constraining influence whereby more membership interlock within a network domain was associated with less future tie creation within it. Given these earlier findings, I posit the following hypothesis:

**Hypothesis #2b:** Greater PON formation will occur in network domains where less membership interlock occurred among active PONs in the previous year.

Lastly, past research indicates that the duration for which an actor is committed to some existing PON(s) may affect the frequency with which that actor forms or participates in additional networks. Organizational actors operate with finite amounts of resources (e.g., money, staff time) that can prompt them to make strategic decisions about which PONs they join (Agranoff and McGuire 2001; Angst et al. 2022; Nowell and Albrecht 2024), particularly given that participating in an additional PON could be accompanied by diminishing returns on the resources they contribute (Scott and Thomas 2017). Nowell and Albrecht (2024), for example, find that as time passes and new PONs form, the members of existing PONs may leave these networks to join new ones. Owing to the results of these earlier works, I advance my final hypothesis:

**Hypothesis #2c:** Less PON formation will occur in network domains where the average duration of PONs formed in the prior year is longer.

## Research Case, Data, and Analytical Approach

### Research Case

My research case consists of PONs formed by organizational stakeholders in Oregon watersheds for some shared purpose(s), collaborative watershed restoration under the Oregon Plan for Salmon and Watersheds (OPSW) (Liu et al. 2025; Mehdi and Nabatchi 2023; OWEB 2026). Over time, Oregon has taken collaborative approaches to problem-solving in multiple policy domains (Yoon et al. 2022), with watershed issues being no exception (e.g., Margerum 2011; Mehdi and Nabatchi 2023). In the final decades of the 1900s, public officials began passing policies responding to the proliferation of environmentally-harmful behaviors (e.g., overfishing, industrial pollution), their effects of watershed ecosystems, especially salmonid species, and a lack of legislation addressing these consequences (Arha et al. 2003; Habron 2003). The Governor’s Watershed Enhancement Board (GWEB) was created in 1987 to carry out programs offering resources (e.g., technical expertise, funding) to encourage governmental and nongovernmental stakeholders working together for watershed management and restoration (Arha et al. 2003). In 1993, the state legislature developed funding incentives for the creation of watershed health programs in watersheds within which voluntary-formed watershed councils (WCs) had formed. Later, in 1995, lawmakers continued their focus on facilitating watershed management through WCs by introducing formal guidance and criteria for county governments when forming WCs, including provisions for cross-sectoral membership on council boards (Arha et al. 2003; Habron 2003).

Notably, in 1997, Oregon policymakers enacted the OPSW, which coupled the existing laws for water resource management (e.g., regulations for water, wildlife, and minerals) with incentives (e.g., grant funding) for voluntary collaborations, such as restoration projects, between governmental and nongovernmental stakeholders (Arha et al. 2003; Habron 2003; OWEB 2026). Although earlier policies promoted local-level watershed management, the OPSW marked a renewed commitment to community-based approaches by more strongly positioning, and for some situations, outright directing WCs and soil and water conservation districts (SWCDs) to serve as key actors in these collaborative efforts (Arha et al. 2003). Under the OPSW, a variety of stakeholders have convened to form thousands of on-the-ground restoration projects in which participants pool financial, technical, and human (i.e., in-person efforts) to perform activities (e.g., tree planting, erosion control) and achieve shared goals (e.g., increase the amounts of native plant species, enhance streambank stabilization) (OWEB 2026).

In this study, I treat restoration projects with three or more members as PONs forming to accomplish agreed-upon goals through cross-sector collaboration by organizational stakeholders from public, nonprofit, civic, or private sectors. The projects studied in this article meet the criteria of PONs set by Nowell and Milward (2022) as they contain three or more actors working together under some shared affiliation (i.e., project name) to complete shared purposes (i.e., project goals). Unlike many of the PONs examined in past scholarship (e.g., Nowell and Albrecht 2024; Nowell et al. 2019), though, these project-based PONs often operate for shorter periods of time (e.g., 1-2 years) rather than for several or more years. Additionally, the members of these PONs are working together to achieve goals for hands-on, watershed restoration that facilitate policy implementation rather than for purposes of policymaking or interorganizational planning like networks in prior studies (e.g., Lemaire et al. 2024; McLaughlin et al. 2022; Scott and Thomas 2017).

To identify the network domains within which these project-based PONs form, I utilize geospatial data on HUC8 watershed subbasins. While prior PON scholarship has used county jurisdictions to bound network domains (e.g., Nowell and Albrecht 2024; Nowell et al. 2019), bounding decisions are ultimately made by the researcher after consideration of the issue and/or context of their study (Nowell and Milward 2022; Taylor and Nowell 2024). Water resources and issues are not usually confined exclusively to a particular county nor its stakeholders, leading watershed boundaries, particularly HUC8 geological boundaries, to be used to study watershed collaborations (Liu et al. 2025; Scott 2016). HUC8 boundaries typically cover areas ranging from several hundred to a few thousand square miles and are thereby sizable enough to capture meaningful hydrological patterns and governance structures, but specific enough to avoid aggregating across starkly different ecological or administrative contexts.

### Data

In this paper, all data on PONs for collaborative watershed restoration occurring under the OPSW comes from the Oregon Watershed Restoration Inventory (OWRI). The OWRI is maintained by the Oregon Watershed Enhancement Board (OWEB), formerly GWEB, as a publicly-accessible resource for information on over 20,000 restoration projects completed since 1995. Some projects received governmental funding and are required to report information about their work. Most recorded projects, however, are voluntarily reported to OWEB, with submitted information reviewed for accuracy by agency staff. For each project, the OWRI contains data including participant information, in-kind contributions, goals, and duration. Most participants are organizational actors (e.g., public agencies, conservation groups, firms). Private landowners are also recorded as participants; however, these entries are usually anonymized. These participant records are excluded from this study as the lack of unique identifiers prevents interlocks of these actors from being examined. Thus, inter-PON dynamics regarding network members, like membership interlock, are examined at the organizational level. For this study, I use the OWRI to collect data on the number of new PONs in each watershed (i.e., network domain) in each year, a PON’s participants, acquired resources (i.e., monetary funding, in-kind contributions), and duration.

Additionally, I gathered data on network domains’ water quality using the Oregon Water Quality Index (OWQI). The OWQI was developed by the Oregon Department of Environmental Quality (DEQ) to assess water quality at monitoring stations across the state’s watersheds. The OWQI is a useful measure of water quality given its aggregation of eight common measures of water resources’ health – (1) water temperature which is critical for maintaining and restoring salmonid populations and can be affected by human activities (e.g., water from power plants or dams, use of water for irrigation); (2) pH which has direct effects on the health and survival of aquatic organisms; (3) the amount of dissolved oxygen within water as aquatic life require sufficient oxygen in their habitats; (4) a measure of biochemical oxygen demand or the amount of dissolved oxygen needed by aerobic organisms in a body of water; (5) the extent of total solids in a water column which reflects the amount of sediment within water; (6 and 7) nitrogen and phosphorus as both nutrients are essential for plant growth and can be affected by human activities (e.g., industrial pollution, agricultural runoff); and (8) the presence of *Escherichia coli* (*E. coli*) bacteria which can adversely affect human health – into a single score (Brown and Maloney 2024). While the OWQI simplifies the health of very complex watershed systems, its eight individual measures nevertheless capture multiple key conditions of water resources within Oregon’s watersheds. Moreover, the DEQ views this simplicity as a strength as it enables the communication of information about the overall water quality to stakeholders (e.g., policymakers, residents) in an accessible manner (Brown and Maloney 2024; Cude 2001; Liu et al. 2025).

### Analytical Approach

**Fig. 3:**
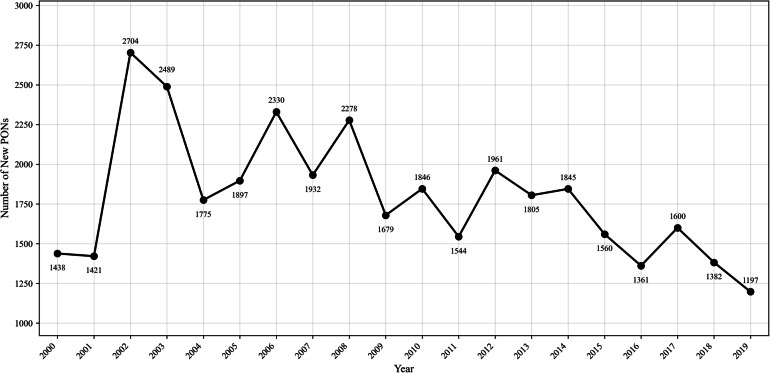
Formation of project-based watershed restoration PONs in Oregon between 2000 and 2019

The OWRI documents far fewer restoration PONs occurring prior to 1997 when the OPSW was implemented. Thus, to examine potential factors affecting PON formation, I scope my analysis to examine a twenty-year period from 2000 to 2019. In selecting this time period, I exclude any pre-OPSW PONs along with those forming in the earliest years of the OPSW’s implementation, when the voluntary participation dynamics of these networks’ formation may have still been developing. With this study period, I also avoid analyzing projects created in 2020 and beyond, for which participation may have been affected by the COVID-19 pandemic. Figure 3 traces statewide PON formation between 2000 and 2019.

Using the OWRI and the DEQ’s OWQI data, I construct a longitudinal dataset of PON formation, network characteristics, and water quality for 52 network domains (i.e., watershed subbasins) between 2000 and 2019. Although Oregon contains 92 subbasins, only 68 subbasins contain an OWQI monitoring station. Furthermore, of these 68 subbasins, OWQI data for all examined years is available for only 52 subbasins. With this dataset, I perform a series of regression analyses to identify factors associated with PON formation. The dependent variable in these analyses measures the number of project-based PONs forming in a domain in a given year. Since my dependent variable is a non-negative integer, rather than a continuous measure, an ordinary least squares regression is inappropriate given that such a model assumes a continuous, normally distributed dependent variable. As such, I use a count data model which does not involve this assumption, but instead models the outcome (e.g., the number of PONs forming in a watershed) as resulting from a discrete probability distribution bounded at zero.

The baseline count data model is a Poisson regression, which assumes that the conditional mean and variance of a count variable (e.g., new PONs) equals its conditional mean (i.e., assumes equidispersion). Count data often violates this assumption, though, exhibiting overdispersion where the variance exceeds the mean. In my dataset, overdispersion occurs, with the variance-to-mean ratio of approximately 34.0, far exceeding the value of 1.0 expected under the Poisson distribution. Negative binomial regressions, however, allow for overdispersion through the inclusion of an alpha parameter, allowing the variance to exceed the mean. Therefore, given the overdispersion in my dependent variable, I utilize negative binomial regressions to estimate the expected count of new PONs within a network domain in a given year as a function of covariates, including key independent variables corresponding to the hypotheses advanced earlier.

To examine whether water resource conditions may be a factor in PON formation, I include a variable measuring the average water quality in a network domain as indicated by the OWQI (*Average OWQI Score*_*t-2*_). I construct this variable by first using geodata for each OWQI monitoring station to match each station to a subbasin. Then, for each subbasin, I calculate the average OWQI score across all samples taken at a station in a given year to create station-year averages. Finally, I average the station-year scores across all of a subbasin’s monitoring stations to arrive at a subbasin-year average. I also lag this variable by two years on account of the DEQ’s annual water quality report being published sometime within the first five months of the year after OWQI data is collected. This two-year lag allows for sufficient information processing time by watershed stakeholders.

Given existing expectations that the activities of policy-prescribed actors are influential in the development of watershed collaborative efforts like the studied project-based PONs, I also include independent variables measuring the number of PONs within a domain in which WCs (*WC Activity*_*t-1*_) and SWCDs (*SWCD Activity*_*t-1*_) participated during the previous year. The OPSW identifies both actors as key for community-based collaborative watershed restoration. A unique variable was created for each actor, although WCs and SWCDs have somewhat similar missions regarding watershed management and restoration, these entities form through different governmental processes that contribute to markedly different institutional forms and jurisdictional scopes (i.e., WCs operate across counties while SWCDs generally operate at the county level). These actors are thus distinct from other policy actors in the OPSW, like public agencies, that generally operate across larger jurisdictions and address a broader bandwidth of issues. Additionally, as community capacities (e.g., human, social, and financial capital) are found to potentially influence watershed collaborations’ formation, I include an independent variable, (*PON Resources*_*t-1*_), to capture the aggregate amount of cash or in-kind resources involved in a domain’s PONs in the prior year.

Alongside these measures of network domains’ environmental features, I include independent variables representing inter-PON dynamics within domains. I include a variable reflecting the number of PONs formed in a domain in the previous year (*Network Domain Saturation*_*t-1*_). I also include a variable measuring the average membership interlock portfolio (i.e., the number of PONs in which an actor simultaneously participates) among the participants of a domain’s PONs in the previous year (*Average Membership Interlock Portfolio*_*t-1*_). This variable was created by identifying all organizational actors participating in PONs created or already ongoing in the prior year and then calculating the average number of PONs per actor. A value of 2 for this variable, for example, would indicate that, on average, an actor was a member of two PONs in the previous year. In creating this variable, I exclude the portfolios of five public agencies: the Bureau of Land Management, the Oregon Department of Fish and Wildlife, the Oregon Department of Forestry, the Oregon Water Resources Department and OWEB^1^. I exclude these actors as they frequently participate in PONs, given their bureaucratic roles in the OPSW. These actors’ interlock portfolios, therefore, are not likely to be representative of the portfolios of other watershed stakeholders (e.g., civic groups, sporting clubs, ranchers) who are more prone to the resource constraints typically associated with membership interlocks. For my last key independent variable, I include a variable capturing the average duration of PONs forming within a domain in the prior year (*Average PON Duration*_*t-1*_) to investigate the potential influence of the extent of actors’ commitments to existing PONs.

In addition to these key explanatory variables, I include a control variable for the number of PONs occurring in neighboring network domains in the previous year (*Neighboring Domains’ PONs*_*t-1*_) to account for any influence of PON formation outside, but near a domain. This variable captures possible spatial diffusion effects whereby PON formation in adjacent domains may spill over into the focal domain. Lastly, I include domain (i.e., subbasin) and year fixed effects to address any trends in PON formation within specific domains or years.

To account for skewed distributions and to enable interpretation of effects in percentage changes, I applied log transformations to *WC Activity*_*t-1*_, *SWCD Activity*_*t-1*_, *PON Resources*_*t-1*_, *Network Domain Saturation*_*t-1*_, *Average Membership Interlock Portfolio*_*t-1*_, and *Neighboring Domains’ PONs*_*t-1*_. For variables that could have a value of zero, a small constant (0.01) was added before log transformation to retain observations while minimizing distortion of any underlying relationships. Count models, by default, use a log link function to model the relationship between predictors and expected values of the dependent variable’s count data. Finally, it should be noted that *PON Resources*_*t-1*_, *Network Domain Saturation*_*t-1*_, and *Average Membership Interlock Portfolio*_*t-1*_ exhibited high variance inflation factors ( > 10) when included in regression models simultaneously, indicating problematic multicollinearilty. To address this issue and avoid inflated standard errors and unstable coefficient estimates, I estimate three separate model specifications, each including one these three variables while holding the remaining covariates constant. This approach allows for direct testing of my hypotheses while maintaining reliable inference. Each regression model equation is written out next.

***PON Resources Model:***

$$\begin{array}{lll}{\rm{Log}}\left(Y\right)&=&{{\rm{\beta }}}_{0}+{{\rm{\beta }}}_{1}{{AverageOWQIScore}}_{t-2}+{{\rm{\beta }}}_{2}{{WCActivity}}_{t-1}\\&&+\,{{\rm{\beta }}}_{3}{{SWCDActivity}}_{t-1}+{{\rm{\beta }}}_{4}{\log ({PONResources})}_{t-1}\\&&+\,{{\rm{\beta }}}_{5}{{AveragePONDuration}}_{t-1}\\&&+\,{{\rm{\beta }}}_{6}\log \left({{NeighboringDomainsPONs}}_{t-1}\right)\\&&+\,{\rm{\mu }}{Domain}+\lambda {Year}+e\end{array}$$

***Network Domain Saturation Model:***

$$\begin{array}{lll}{\rm{Log}}\left(Y\right)&=&{{\rm{\beta }}}_{0}+{{\rm{\beta }}}_{1}{{AverageOWQIScore}}_{t-2}+{{\rm{\beta }}}_{2}{{WCActivity}}_{t-1}\\&&+\,{{\rm{\beta }}}_{3}{{SWCDActivity}}_{t-1}\\&&+\,{{\rm{\beta }}}_{4}{\log ({NetworkDomainSaturation})}_{t-1}\\&&+\,{{\rm{\beta }}}_{5}{{AveragePONDuration}}_{t-1}\\&&+\,{{\rm{\beta }}}_{6}\log \left({{NeighboringDomainsPONs}}_{t-1}\right)\\&&+\,{\rm{\mu }}{Domain}+\lambda {Year}+e\end{array}$$

***Membership Interlock Model:***

$$\begin{array}{lll}{\rm{Log}}\left(Y\right)&=&{{\rm{\beta }}}_{0}+{{\rm{\beta }}}_{1}{{AverageOWQIScore}}_{t-2}+{{\rm{\beta }}}_{2}{{WCActivity}}_{t-1}\\&&+\,{{\rm{\beta }}}_{3}{{SWCDActivity}}_{t-1}\\&&+\,{{\rm{\beta }}}_{4}{\log ({AverageMembershipInterlockPortfolio})}_{t-1}\\&&+\,{{\rm{\beta }}}_{5}{{AveragePONDuration}}_{t-1}\\&&+\,{{\rm{\beta }}}_{6}\log \left({{NeighboringDomainsPONs}}_{t-1}\right)\\&&+\,{\rm{\mu }}{Domain}+\lambda {Year}+e\end{array}$$

## Results

I begin reporting the study’s results by briefly reviewing descriptive statistics (shown in Table 1) for the dependent and key independent variables. In the sample’s “average” network domain, approximately 29 PONs formed each year, with a standard deviation (SD) of roughly 32 PONs. Within the “average” domain, around 29 PONs formed in the prior year (SD ~ 33 PONs). Of these previously-formed PONs, the average network lasted approximately thirteen months (SD ~ ten months). Around nine of the active (i.e., new or ongoing) PONs in the domain in the prior year involved a WC (SD ~ eleven PONs), and roughly eleven had a SWCD as a participant (SD ~ eleven PONs). Furthermore, among these active networks, actors’ average interlock portfolio was roughly two PONs (SD ~ two PONs). Additionally, PONs forming in the previous year possessed resources with an aggregate average value of $653,443.43 (SD = $ 1,728,040.38). Finally, the “average” domain received an average OWQI score of 80.60 (SD = 16.51), indicating “Fair” water quality two years prior.

**Table 1:** Descriptive statistics of watershed network domains

	New PONs	Average OWQI Score _*t-2*_	WC Activity_*t-1*_	SWCD Activity_*t-1*_	PON Resources_*t-1*_	Network Domain Saturation_*t-1*_	Average Membership Interlock Portfolio_*t-1*_	Average PON Duration_*t-1*_ (*months*)	Neighbor Domains’ PONs_*t-1*_
**Mean**	28.61	80.60	9.14	10.81	$653,443.43	28.68	1.89	12.58	149.54
**SD**	32.33	16.51	11.18	11.05	$1,728,040.38	33.11	1.81	9.91	113.14
**25th** **Percentile**	9.00	77.79	2.00	3.00	$70,826.75	9.00	1.03	5.63	72.75
**75th** **Percentile**	36.00	91.05	11.51	14.50	$664,855.75	36.00	2.25	17.97	199.00

Moving into the results of the regression analyses, these results are reported in Table 2. The table’s first column contains results for the model containing *PON Resources*_*t-1*_. The second column reports results pertaining to the model including *Network Domain Saturation*_*t-1*_. The third column shows results for the model with *Average Membership Interlock Portfolio*_*t-1*_.

**Table 2:** Negative binomial regression results for PON formation

	PON Resources	Network Domain Saturation	Membership Interlock
**Average OWQI Score**_**t-2**_	−0.0056 (0.0045)	−0.0050 (0.0045)	−0.0050 (0.0045)
**WC Activity**_**t-1**_	0.0064 (0.0228)	0.0020 (0.0221)	0.0056 (0.0227)
**SWCD Activity**_**t-1**_	0.0386 (0.0783)	0.0311 (0.0759)	0.0350 (0.0309)
**PON Resources**_**t-1**_	0.0232** (0.0101)		
**Network Domain Saturation**_**t-1**_		0.0679*** (0.0216)	
**Average Membership Interlock Portfolio**_**t-1**_			0.0848*** (0.0317)
**Average PON Duration**_**t-1**_	−0.0088*** (0.0036)	−0.0093*** (0.0034)	−0.0088** (0.0035)
**Neighboring Domains’ PONs**_**t-1**_	0.0558 (0.0783)	0.0456 (0.0759)	0.0472 (0.0763)
**Constant**	2.2653*** (0.3551)	2.4226*** (0.3520)	2.5053*** (0.3587)
**Alpha**	0.4302*** (0.0498)	0.4253*** (0.0494)	0.4279*** (0.0496)
**Domain FE**	X	X	X
**Year FE**	X	X	X
**Metric**			
**Log-Likelihood**	-4102.7897	-4098.6822	-4100.9661
**AIC**	8361.5793	8353.3644	8357.9322
**BIC**	8747.4434	8739.2286	8743.7963
**Observations**	1040	1040	1040

Standard errors are reported in parentheses. *Significance level *p* < 0.1. **Significance level *p* < 0.05. ***Significance level *p* < 0.01

My first research question asked whether influential factors (i.e., water resource conditions, policy actor activities, network domain resource levels) in the development of PONs for collaborative policymaking or interorganizational planning are also relevant in the formation of PONs engaging in action-level collaboration for hands-on policy implementation. In all three models, the coefficient for *Average OWQI Score*_*t-2*_ is negative, but not statistically significant. The coefficients for *WC Activity*_*t-1*_ and *SWCD Activity*_*t-1*_ are positive, but also not significant. In the first model including *PON Resources*_*t-1*_, however, this variable’s coefficient is positive and statistically significant. Since this variable was log-transformed, its coefficient suggests that a 1% increase in resource contributions to PONs formed in a domain in one year is associated with a 0.023% increase in PON formation in the following year.

My second research question asked whether inter-PON dynamics (i.e., domain saturation, membership interlock, and network duration) affect the formation of new PONs. Regarding *Network Domain Saturation*_*t-1*_, the second model reports a positive and statistically significant coefficient. After accounting for the variable’s log transformation, this coefficient indicates that a 1% increase in domain saturation in a given year is associated with a 0.068% increase in the subsequent year’s PON formation. In the third model, a positive and significant coefficient is also found for *Average Membership Interlock Portfolio*_*t-1*_. As this variable was also log transformed, its coefficient implies that a 1% increase in the average membership interlock portfolio of a domain’s actors (excluding key public agencies) is associated with a 0.085% increase in PON formation in the next year. Meanwhile, for *Average PON Duration*_*t-1*_, this variable’s coefficients are negative and significant across all three models. Since this variable was not log transformed, these coefficients suggest that a 1-month increase in the average duration of PONs formed in a domain one year is associated with a 0.88–0.93% decrease in PON formation in the next.

Lastly, in all three models, the coefficient for *Neighboring Domains’ PONs*_*t-1*_ was positive but not statistically significant. The lack of a significant relationship between this variable and PON formation suggests that formation spillover effects do not occur at the domain level, whereby increased formation in one domain would influence formation in an adjacent domain. These results, however, do not entirely rule out the possibility of spatial autocorrelation where the models’ error terms would be correlated across space, potentially biasing coefficient estimates. To more comprehensively address this concern, I conduct Moran’s I tests with a queen contiguity spatial weights matrix to assess each model for spatial autocorrelation. The test statistic for each model is reported in Table 3. This test statistic is only statistically significant (*p* = 0.044) for the membership interlock model and indicates weak spatial autocorrelation (0.169). Moderate spatial autocorrelation would be signaled by a value greater than 0.30. Given the small magnitude of this statistic along with the high statistical significance of the interlock coefficient (p = 0.007), substantive interpretations regarding this variable remain robust. Moreover, the absence of statistical significance for *Neighboring Domains’ PONs*_*t-1*_ suggests limited cross-watershed spatial spillovers.

**Table 3:** Results of Moran’s I test for spatial autocorrelation

Model	Moran’s I	P-value
**PON Resources**	−0.0413	0.420
**Network Domain Saturation**	−0.0572	0.364
**Membership Interlock**	0.1686**	0.044

*Significance level *p* < 0.1. **Significance level *p* < 0.05. ***Significance level *p* < 0.01

## Discussion

Across the two decades of PON formation that this study analyzes, it generates novel knowledge on the influences on the development of these networks. In particular, my findings offer new insight into potential factors shaping the creation of PONs for cross-sector collaborations (i.e., actors from different organizations and sectors jointly determining a course of action, pooling resources, and working together to achieve shared goals) that facilitate on-the-ground policy implementation. Networks in which these collaborations occur are becoming increasingly prevalent for tackling complex public problems, especially intricate and interrelated environmental issues like those in watersheds (Liu et al. 2025; Margerum 2008; 2011; Nowell and Milward 2022).

The study’s first research question sought to assess whether relevant factors in the formation of watershed collaborations, including networks for policy-level (i.e., policymaking) or organizational-level (i.e., interorganizational planning or coordination), are also influential in the formation of action-level (i.e., hands-on policy implementation) networks. Hypothesis #1a predicted that when water resource conditions were more severe (in this analysis, when a network domain received a lower water quality score two years prior), increased PON formation would occur in the examined year. No statistically significant results were found in relation to this hypothesis, which could suggest that the factors of resource quality or problem severity may not be as relevant for the development of project-based PONs focused on hands-on addressment of public problems through activities like tree planting or erosion control. These results, however, do not rule out resource conditions as potential influences on action-level PON formation. It is possible that the variable (i.e., average OWQI scores) used to test this hypothesis is not the most apt measure and that other indicators of watershed health (e.g., the health or abundance of salmonid populations) might be more pertinent to the formation of action-level restoration PONs.

I also do not find statistically significant results regarding Hypothesis #1b, which expected that in domains within which WCs and SWCDs (actors prescribed pivotal roles in policy implementation by lawmakers) participated more extensively in new or ongoing PONs in the prior year, greater PON formation would follow. The lack of significant findings regarding the activity of these policy-prescribed actors does not imply that they are not influential in PON formation, but suggests that their influence could occur through different pathways. Actors like WCs or SWCDs can, for example, engage in programming (e.g., regular meetings, planning forums) that bring stakeholders together and may lay the groundwork for collaboration in project-based PONs (McLaughlin et al. 2022; Scott and Thomas 2017).

Unlike the prior two hypotheses, though, I identify a statistically significant relationship pertaining to Hypothesis #1c, which posited that higher aggregate resource levels (i.e., monetary or in-kind resources) among a domain’s PONs in one year would be positively associated with PON formation in the next year. I find support for this hypothesis, although the magnitude of this relationship is modest in terms of substantive changes to the number of PONs forming in domains. For instance, an increase in a domain’s PONs’ resources from the 25th percentile ($70,827) to the 75th percentile ($664,856) is associated with approximately 1.5 additional networks forming in the subsequent year.

Although the influence of a domain’s resource levels on future PON formation may not be sizable, this finding does indicate that resource capacity is salient in the formation of these networks. Furthermore, this result speaks to the importance of practitioners and researchers considering resource dependencies among the PONs in a domain from one point in time to another. For instance, in their investigation of population dynamics within network domains, Nowell and Albrecht (2024), observed that domains containing more networks reached their “carrying capacity” at which point the number of networks within a domain was either maintained or reduced (p. 2228). My finding suggests that, in the context of these action-level networks, a domain’s carrying capacity is not static and that the amount of monetary or in-kind resources contributed to a domain’s PONs in a given year may shape its capacity for network formation in the next.

Resource dependencies and interdependencies within network domains are further explored through analyses addressing my second research question, which asked whether inter-PON dynamics may influence the formation of action-level PONs. Hypothesis #2a anticipated that when a network domain is more saturated by the development of new PONs in one year, fewer PONs will form in the following year. I find support for this hypothesis, although the magnitude of this relationship is also modest. An increase from the 25th percentile (9 PONs) to the 75th percentile (36 PONs) within a domain, for example, would be associated with an additional 2.7 PONs forming in the subsequent year.

Nevertheless, this finding is interesting as it runs counter to expectations advanced by prior scholarship that anticipate more saturated domains experiencing no increase, or even a reduction, in the number of networks in the future. The increased PON formation identified in this study could be a product of the differences in duration and purpose of the examined PONs. These networks are forming to engage in projects for hands-on watershed restoration activities and policy implementation, unlike the policymaking or planning purposes of networks in earlier work. Additionally, these project-based PONs are typically completed within a year or two, unlike other networks in earlier studies, which engage in longer-term (i.e., several years) or continuous operations (e.g., Lemaire et al. 2024; Nowell et al. 2019). The differences of these project-based PONs may diminish the influence of resource constraints created by greater domain saturation and instead, allow the more substantial formation in one year to catalyze formation in the next. This “more PONs beget more PONs” scenario also is suggested by my earlier finding around prior network resource levels within a domain.

Similarly, the results pertaining to Hypothesis #2b and membership interlock also suggest that the inter-PON dynamics of networks for action-level policy implementation may differ from those of networks for policymaking or interorganizational planning. This hypothesis expected that when more extensive membership interlock occurs in a domain, fewer PONs would form in the upcoming year. My results imply the opposite. An increase in a domain’s average membership interlock portfolio (i.e., the number of ongoing or new PONs in which an actor simultaneously participates) is associated with greater future PON formation. A 1-PON increase in a domain’s average interlock portfolio (i.e., shifting from the 25th percentile, 1.03 PONs, to the 75th percentile, 2.25 PONs), is associated with an additional 1.9 PONs in the next year. Thus, this result suggests that broader (i.e., multi-PON) participation by a domain’s actors may spur PON creation rather than constrain it. Once again, these project-based PONs’ differences in duration and purpose, compared to PONs in past research, may contribute to this finding. The shorter-term nature and on-the-ground focus on the studied PONs may lead to co-participation in multiple networks to facilitate relationships (e.g., mutual interests, complementary skills, knowledge sharing) that enable heightened PON formation moving forward.

Interestingly, my findings regarding Hypothesis #2c and PON duration conform to the expectations of existing literature. This hypothesis posited that as the average duration of PONs formed in a domain during a year increased, fewer PONs would form in the following year. I find support for this hypothesis, with my results suggesting that the time commitments of previously-formed PONs may influence subsequent PON development. For instance, an increase in a domain’s average PON duration from the 25th percentile (5.63 months) to the 75th percentile (17.97 months) is associated with approximately 3.1 fewer PONs forming in the next year. A more probable 6-month increase in average duration is associated with roughly 1.5 fewer PONs.

Thus, within the examined network domains, higher levels of prior PON resources, greater domain saturation, and more extensive membership interlock were associated with higher rates of future PON formation, while longer durations of previously-formed PONs were associated with less subsequent PON development. Taken together, these findings underscore the salience of access to, and provision of, resources (e.g., grant funding, network participants’ in-kind contributions) for PONs in network domains. While more research on the population dynamics of PONs is needed, my findings also suggest potential ramifications of actors’ decisions around network participation on future network formation. More specifically, attention should be paid not just to decisions of whether actors participate, but how actors participate (e.g., how they contribute to PONs’ resources, how many PONs they join) as the breadth of an actor’s PON participation within a domain appears to be a meaningful factor in the creation of PONs for on-the-ground policy implementation.

Additionally, as all three variables measuring inter-PON dynamics were meaningfully associated with changes in PON formation, but not all had relationships in the directions anticipated by past studies, my results motivate further inquiry into the relationships of actors within and across PONs, as these dynamics could have meaningful ramifications for the networks that do (or do not) form. Furthermore, my results emphasize the need for more consideration of differences in PONs’ constitutive dimensions. I analyze PONs operating, on average, for a little more than a year. This duration differs substantially from the duration of many networks in earlier PONs scholarship, which often operate for at least several years. The shorter operating times of these project-based PONs may induce participatory dynamics that diverge from those in other previously-examined networks. For example, as these PONs do not usually constitute multi-year commitments of time and resources, actors may encounter a different decision-making calculus when considering whether to join another PON as opposed to if they were contemplating participation in one posing more extensive commitments.

Beyond these durational differences, these project-based PONs differ from many of those in prior research as their members collaborate at the action level. That is, these networks are not forming to design policies (i.e., policy-level networks) or create multi-organizational strategies (i.e., organizational-level networks), but rather, to carry out activities intended to produce outputs directly in watersheds (Margerum 2011). The action-level work of these PONs may contribute to some of this study’s unexpected findings, as what influences the development of policy- or organizational-level networks may be fundamentally different from what shapes action-level PON formation, given their functionally-distinct purposes.

Relatedly, PONs with different functional outlooks may demand different extents of participant commitment (e.g., time, resources). Project-based PONs could require less time and resources from their members, allowing for less uncertainty in how these actors make decisions about future PON participation. This reduced uncertainty, in turn, may enable actors to maintain larger interlock portfolios (i.e., to participate in more PONs simultaneously). Both scenarios are observed in this study. The positive association between the extent of membership interlock in a domain and the number of PONs formed in the subsequent year suggests that stakeholders being more extensively involved in PONs in the prior year may actually amplify PON formation in the next. One potential explanation for this finding, however, is that different sets of actors are participating in interlocked PONs in one year than those developing new PONs in the next. A study of the longitudinal participation of PON members would be a fruitful avenue for future research.

## Conclusion

Through its investigation of the formation of watershed restoration networks, this study contributes to our understanding of purpose-oriented networks, particularly those engaging in action-level, cross-sector collaboration to facilitate the implementation of public policies. Despite action-level networks often being vital for responding to public problems, especially those that governments have not, or cannot, effectively address on their own (Margerum 2008), these networks have received less attention in existing research. In response to this research gap, this paper provides novel insight into the factors that can influence these networks’ formation. Notably, I find that greater network domain saturation, more extensive overlapping PON participation by stakeholders (i.e., larger membership interlock portfolios), and greater resource levels within a domain’s new PONs may catalyze future network formation. My results also suggest that longer operating durations of PONs formed in one year may diminish network development in the next. Collectively, these findings highlight the importance of considerations for resource provision mechanisms and network interdependencies by policymakers and practitioners when designing and carrying out policies relying on cross-sector arrangements like PONs.

Alongside these practical implications, this article expands PON scholarship by analyzing networks for policy implementation at the action level, as many of the existing studies of networks for environmental management focus on those for policymaking or interorganizational planning (e.g., McLaughlin et al. 2022; Scott and Thomas 2017). Moreover, this investigation serves as an application of the understudied external perspective of networks (e.g., Nowell et al. 2019; Rethemeyer and Hatmaker 2008). Through this application, my study demonstrates how examinations of a network’s broader environment (i.e., its network domain) are necessary for comprehensively understanding networks’ development and performance. The contrast that emerges between some of my findings and those in earlier work highlights the need for further inquiry around the constitutive dimensions of PONs (Carboni et al. 2019) and the ramifications of differences in these dimensions. Ultimately, with policymakers increasingly relying on PONs to respond to complex public problems, it is imperative that practitioners and researchers are equipped with knowledge on how these networks form and what amplifies or diminishes their formation.

## Footnotes

1 An interlock variable was also created that did not exclude these public agencies from its calculation. The study’s findings regarding membership interlock are robust across both specifications.
